# Improved survival with radiotherapy in hepatocellular carcinoma with major vascular invasion: A propensity‐matched analysis of Surveillance, Epidemiology, and End Results database

**DOI:** 10.1002/cam4.1937

**Published:** 2019-01-18

**Authors:** Qiuyan Lin, Xiaoquan Huang, Canmei Zhong, Tiancheng Luo, Xiaoqing Zeng, Shiyao Chen

**Affiliations:** ^1^ Department of Gastroenterology, Union Hospital Fujian Medical University Fuzhou China; ^2^ Department of Gastroenterology, Zhongshan Hospital Fudan University Shanghai China

**Keywords:** hepatocellular carcinoma, major vascular invasion, propensity score‐match, radiotherapy, Surveillance, Epidemiology, and End Results

## Abstract

**Objectives:**

Hepatocellular carcinoma (HCC) associated with major vascular invasion is an advanced stage disease with an extremely poor prognosis and low survival rate. Our study evaluated the survival benefit of radiotherapy (RT) in HCC patients with major vascular invasion through Surveillance, Epidemiology, and End Results database.

**Methods:**

We analyzed 3181 HCC patients with major vascular invasion cases diagnosed from 2004 to 2013. Patients (N = 308) who underwent RT and patients (N = 2873) who did not receive RT were compared. We successfully analyzed patients using propensity score matching (PSM). Kaplan‐Meier and Cox‐regression analyses were applied to assess prognosis.

**Results:**

The median survival time in radiation‐treated group was longer compared to the control group (7 months vs 3 months; *P* < 0.001) in the overall sample and 3 months longer compared to the control group (7 months vs 4 months; *P* < 0.001) in a PSM cohort. Cox‐regression analyses showed that radiation‐treated patients in propensity‐matched sample had a significantly lower risk of mortality (HR: 0.625, 95% CI: 0.522‐0.749, *P* < 0.001) compared with untreated patients. The radiation‐treated groups had better survival rate than untreated group. Subgroup analysis revealed that the survival time of patients in radiation‐treated group was significantly longer than that in the untreated group (*P* < 0.001 and *P* = 0.026, respectively). The subgroup analysis also revealed that RT provides a survival benefit regardless of race, marital status, and tumor size after PSM.

**Conclusions:**

Radiotherapy provides improves survival in HCC patients with major vascular invasion, especially for tumor(s) confined to one lobe and not on surface of liver.

## 1 INTRODUCTION

According to GLOBOCAN 2012 of the International Agency for Research on Cancer estimates, primary liver cancer is the fifth most frequently diagnosed cancers and second most common cause of cancer‐related deaths worldwide among men. The most frequent liver cancer in about 70%‐90% of patients is HCC.^1^, ^2^ Patients with HCC are prone to vascular invasion and accompanied by dismal prognosis,^3^, ^4^ which frequently arises in the portal vein, the hepatic/cava vein branches, or seldom the hepatic arteries.^5^ In particular, approximately 30%‐40% cases of advanced HCC are accompanied by portal vein tumor thrombosis (PVTT).^6^, ^7^

More pressingly, according to Barcelona Clinic for Liver Cancer (BCLC) Staging System, HCC associated with major vascular invasion is defined as an advanced stage, with an extremely poor prognosis and dismal survival rate.^8^, ^9^ Regular surveillance can help in the early detection of the disease in patients who are at risk, but many patients are already in the intermediate or advanced stage when diagnosed.^10^

Although surgical resection, transarterial chemoembolization (TACE), radiofrequency ablation and liver transplantation have significantly improved the survival rate of patients with HCC, it is prone to tumor recurrence, and the prognosis of advanced HCC still remained unsatisfactory.^11^, ^12^, ^13^ Sorafenib significantly improved the median survival and the time to radiologic progression by nearly 3 months longer for patients with advanced HCC in a multicenter, phase III, placebo‐controlled trial.^14^ However, the application of sorafenib was limited because of grade 3/4 adverse events such as diarrhea, fatigue, and hand‐foot skin reaction, and discontinuation or dose reduction of sorafenib are frequently performed.^14^, ^15^, ^16^ Although recent studies suggested that surgical resection of the liver improved the survival in patients with PVTT or hepatic vein invasion (HVTT), not all patients are eligible for this surgery.^17^, ^18^

Thus far, although more different treatment strategies have been utilized, the prognosis of patients accompanied by advanced HCC associated with major vascular invasion still remained poor. Therefore, more effective treatment to improve the prognosis of patients is urgently required. Radiotherapy (RT) is reported to improve the survival of patients with HCC,^19^ but studies on HCC with major vascular invasion were rarely reported and lacked large sample data. Hence, this study aimed to evaluate the survival benefit of RT in HCC patients with major vascular invasion based on the Surveillance, Epidemiology, and End Results (SEER) database.

## 2 MATERIALS AND METHODS

### 2.1 Study population

We obtained data from the SEER database of the National Cancer Institute (NCI) and performed a retrospective cohort study with cases diagnosed from 2004 to 2013. SEER is an open‐access cancer database of the United States from 18 population‐based cancer registries. SEER currently collects and publishes cancer incidence and survival data covering approximately 28% of the US population and is representative of demographic data.^20^ We acquired patients diagnosed with HCC from SEER database in accordance with International Classification of Diseases for Oncology, 3rd Edition (ICD‐O‐3), histology codes 8170 through 8175 for HCC, and site code C22.0 for liver, not included mixed HCC.^21^ We included patients with HCC extension to vascular invasion (code 630, 635, 660). According to Collaborative Stage Data Collection System Coding Instructions, Part II, Version 02.05, Effective January 1, 2014, major vascular invasion is considered as major branch(es) of portal or hepatic vein(s). Code 630 is major vascular invasion with single tumor (confined to one lobe) or multiple tumor(s) (confined to one lobe and not on the surface of the liver). Code 635 is major branch(es) of portal or hepatic vein(s) plus multiple nodules/tumors in more than one lobe of liver or on the surface of parenchyma. In addition to major branch(es) of the portal and hepatic vein, major vascular invasion of the study also included invasion of the hepatic artery and the inferior vena cava (Code 660). Patients with multiple primary malignant tumors were excluded. HCC patients with less than one month of survival or whose survival data were unavailable were excluded. We excluded patients who underwent surgical resection, such as resection of primary cancer lesions, resection of lymph nodes, or metastatic lesions. To identify control patients, we excluded patients who were unavailable for radiation, and had refused the recommended radiation, but unknown if administered. Follow‐up time of the patients was from HCC diagnosis till death or end of the follow‐up period.

### 2.2 Propensity score matching (PSM)

The purpose of this article was to compare the benefits of RT for HCC patients with vascular invasion. This was an observational study and so the radiation assignment was not random. Some crucial covariates of the patients in the active treatment and control groups were heterogeneous and possibly affected the outcomes. Therefore, we further compared the survival rate between the radiation‐treated and untreated cohorts by using the 1:1 nearest neighbor matching by univariate analysis, setting the caliper as 0.02. The PSM process has been applied to minimize selection bias and approximately balanced the baseline covariates under analytic settings between the groups.^22^

### 2.3 Statistical analysis

The primary endpoint of this study was overall survival (OS). Using chi‐square test, patient characteristics were compared between radiation‐treated and control patients. Covariates in the study included multiple level factors (such as age, gender, race, tumor size, vascular invasion, primary tumor lesion, node, metastasis, stage refer to Derived AJCC Stage Group (6th), differentiation grade, AFP, and degree of fibrosis). The SEER data recorded a small number of tumor lesions that are larger than 20 cm. We thought that these may be inconceivable, and so were included to know the participation statistics partially. The propensity score was used to reduce the selection bias. Kaplan‐Meier analysis was used to estimate OS before and after PSM. We conducted a log‐rank test to compare the survival differences for patients, lesions, and treatment‐related characteristics. For multivariate analysis in the matched population, we constructed a Cox proportional hazards model to identify the predictors of survival. *P* values <0.05 were considered to be statistically significant. All statistical analyses were performed with IBM SPSS Statistics 22.0 (IBM, Armonk, NY, USA).

## 3 RESULTS

### 3.1 Baseline Characteristics

**Figure 1:**
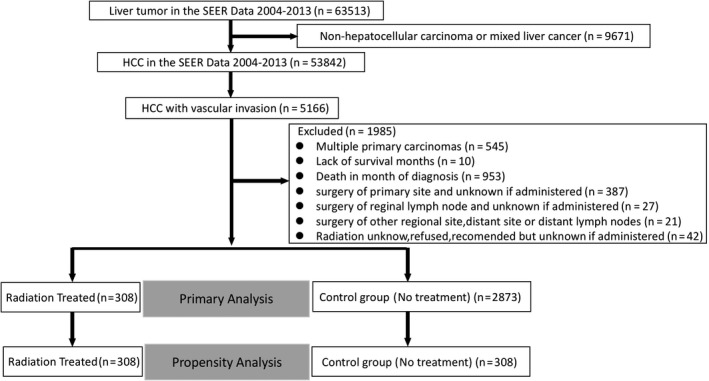
SEER Data extraction and filtering flowchart

From 2004 to 2013, 53 842 patients with newly diagnosed HCC were identified in the SEER data set. Based on the eligibility criteria (described in the study population), a total of 5166 patients diagnosed with advanced HCC associated with major vascular invasion were selected. Of these, 3181 met the inclusion criteria of the study. The radiation‐treated and untreated cohorts included 308 (9.7%) and 2873 (90.3%) patients, respectively (Figure 1). Baseline characteristics of the patients, tumor, and treatment‐related factors are summarized in Table 1.

**Table 1:** Summary characteristics of the overall sample stratified by radiation treatment before and after propensity score matching

Variables	Overall sample (n = 3181)			Propensity‐matched sample (n = 616)		
	Radiation (n = 308)	Control (n = 2873)	*P* value	Radiation (n = 308)	Control (n = 308)	*P* value
Age (y)						
≤65	212 (68.8)	2020 (70.3)	0.59	212 (68.8)	204 (66.2)	0.491
>65	96 (31.2)	853 (29.7)		96 (31.2)	104 (33.8)	
Sex						
Male (%)	251 (81.5)	2393 (83.3)	0.423	251 (81.5)	250 (81.2)	0.918
Female (%)	57 (18.5)	480 (16.7)		57 (18.5)	58 (18.8)	
Marital status						
Married (%)	189 (61.4)	1489 (51.8)	0.003	189 (61.4)	155 (50.3)	<0.001
Single and unmarried (%)	45 (14.6)	659 (22.9)		45 (14.6)	86 (27.9)	
Widowed, divorced, separated (%)	60 (19.5)	596 (20.7)		60 (19.5)	63 (20.5)	
Unknown (%)	14 (4.5)	129 (4.5)		14 (4.5)	4 (1.3)	
Race						
White (%)	224 (72.7)	1852 (64.5)	0.014	224 (72.7)	180 (58.4)	<0.001
Black (%)	43 (14.0)	451 (15.7)		43 (14.0)	46 (14.9)	
Other (%)	41 (13.3)	551 (19.2)		41 (13.3)	82 (26.6)	
Unknown (%)	0 (0.0)	19 (0.7)				
Tumor size						
≤1 cm (%)	0 (0.0)	14 (0.15)	0.008	0 (0.0)	3 (1.0)	0.048
1‐3 cm (%)	26 (8.4)	209 (7.3)		26 (8.4)	18 (5.8)	
3‐5 cm (%)	47 (15.3)	368 (12.8)		47 (15.3)	46 (14.9)	
>5 cm (%)	185 (60.1)	1563 (54.4)		185 (60.1)	168 (54.5)	
Unknown (%)	50 (16.2)	719 (25.0)		50 (16.2)	73 (23.7)	
Vascular invasion						
Code 630 (%)	185 (60.1)	1834 (63.8)	0.23	185 (60.1)	193 (62.7)	0.395
Code 635 (%)	87 (28.2)	685 (23.8)		87 (28.2)	73 (23.7)	
Code 660 (%)	36 (11.7)	354 (12.3)		36 (11.7)	42 (13.6)	
TNM/T						
T3 (%)	272 (88.3)	2519 (87.7)	0.747	272 (88.3)	266 (86.4)	0.467
T4 (%)	36 (11.7)	354 (12.3)		36 (11.7)	42 (13.6)	
Lymph nodes						
N0 (%)	232 (75.3)	2020 (70.3)	0.02	232 (75.3)	209 (67.9)	0.121
N1 (%)	51 (16.6)	457 (15.9)		51 (16.6)	66 (21.4)	
Nx (%)	25 (8.1)	396 (13.8)		25 (8.1)	33 (10.7)	
Distant metastasis						
M0 (%)	213 (69.2)	2038 (70.9)	0.038	213 (69.2)	214 (69.5)	0.891
M1 (%)	85 (27.6)	660 (23.0)		85 (27.6)	86 (27.9)	
Mx (%)	10 (3.2)	175 (6.1)		10 (3.2)	8 (2.6)	
Derived AJCC Stage Group (6th)						
III (%)	213 (69.2)	2038 (70.9)	0.038	213 (69.2)	214 (69.5)	0.891
IV (%)	85 (27.6)	660 (23.0)		85 (27.6)	86 (27.9)	
Unk stage (%)	10 (3.2)	175 (6.1)		10 (3.2)	8 (2.6)	
Grade (differentiated)						
Well (%)	32 (10.4)	186 (6.5)	0.018	32 (10.4)	26 (8.4)	0.088
Moderately (%)	41 (13.3)	335 (11.7)		41 (13.3)	35 (11.4)	
Poorly (%)	32 (10.4)	222 (7.7)		32 (10.4)	19 (6.2)	
Undifferentiated/anaplastic (%)	2 (0.6)	22 (0.8)		2 (0.6)	0 (0.0)	
Unknown (%)	201 (65.3)	2108 (73.4)		201 (65.3)	228 (74.0)	
AFP						
Elevated (%)	233 (75.6)	2198 (76.5)	0.646	233 (75.6)	222 (72.1)	0.568
Within normal limits (%)	36 (11.7)	289 (10.1)		36 (11.7)	39 (12.7)	
Unknown (%)	39 (12.7)	386 (13.4)		39 (12.7)	47 (15.3)	
Fibrosis score						
0‐4 (F0) (%)	13 (4.2)	108 (3.8)	0.136	13 (4.2)	19 (6.2)	0.525
5‐6 (F1) (%)	80 (26.0)	611 (21.3)		80 (26.0)	75 (24.4)	
Unknown (%)	215 (69.8)	2154 (75.0)		215 (69.8)	214 (69.5)	

AJCC, American Joint Committee on Cancer.

### 3.2 Survival before and after PSM

**Figure 2:**
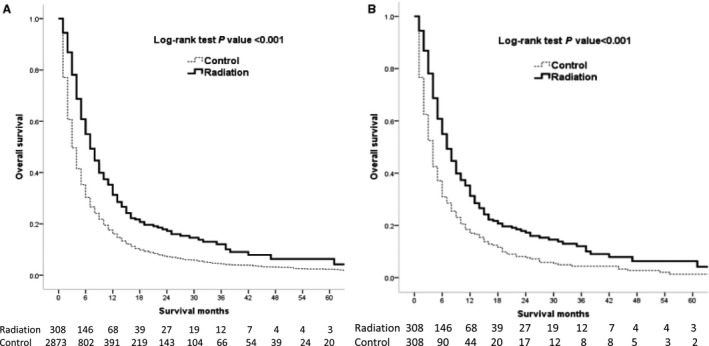
Kaplan‐Meier overall survival curves of radiation‐treated patients vs control before (A) and after (B) propensity score matching

The 3‐month, 6‐month, 1‐year, 3‐year, and 5‐year actuarial survival rates for the radiation treated and untreated groups were 78.1%, 55.0%, 31.3%, 12.1%, and 6.3% vs 49.6%, 30.4%, 16.1%, 4.3%, and 2.3%, respectively (Table 2). The Kaplan‐Meier analyses indicated that the radiation‐treated patients had a significantly better overall survival compared with control patients (*P* < 0.001, Figure 2A). Median survival for patients treated with radiation from the time of HCC diagnosis was 7 (IQR = 4‐15) months, while the median survival of the control groups was 3 (IQR = 2‐8) months.

**Table 2:** The difference of survival rate between the radiotherapy group and the control group before and after the matching

	Before PSM		After PSM	
	Radiation	Control	Radiation	Control
3‐mo actuarial survival (%)	78.1	49.6	78.1	52.5
6‐mo actuarial survival (%)	55.0	30.4	55.0	31.0
1‐y actuarial survival (%)	31.3	16.1	31.3	17.0
3‐y actuarial survival (%)	12.1	4.3	12.1	4.4
5‐y actuarial survival (%)	6.3	2.3	6.3	1.4

After matching the radiation‐treated to control patients with propensity scores, we balanced almost all the available covariates between the groups, while few covariates such as race, marital status, and tumor sizes showed differences. There were 308 radiation‐treated patients and 308 untreated patients matching after excluding the unmatched populations (Table 1). The survival time between the radiation‐treated and the untreated patients also showed significant differences after PSM to balance the covariates (*P* < 0.001, Figure 2B). The 3‐month, 6‐month, 1‐year, 3‐year, and 5‐year actuarial survival for the radiation‐treated group and untreated group after PSM were 78.1%, 55.0%, 31.3%, 12.1%, and 6.3% vs 52.5%, 31.0%, 17.0%, 4.4%, and 1.4%, respectively (Table 2). Median survival of the radiation‐treated patients was 7 (IQR = 4‐15) months and that of the untreated patients was 4 (IQR = 2‐9) months after PSM.

### 3.3 Prognostic factors

Table 3 listed the results of univariate Kaplan‐Meier analysis in the matched population and 6‐month survival rate. Significant differences in the survival rate were seen in covariates with radiation (*P* < 0.001), tumor size (*P* = 0.022), lymph nodes (*P* < 0.001), distant metastasis (*P* < 0.001), AJCC Stage (6th) (*P* < 0.001), and AFP level (*P* < 0.003). The patients with distant metastasis M1 and AJCC stage (6th) IV had worst survival and 6‐months survival rate was only 22.1%.

**Table 3:** Univariate analysis of the matched population (n = 616)

Variables	N (%)	6‐mo survival rate (%)	*P*
Radiation			
Yes	308 (50)	55.0	<0.001
No	308 (50)	31.0	
Age			
≤65	416 (67.5)	43.0	0.459
>65	200 (32.5)	42.6	
Sex			
Male	501 (81.3)	43.2	0.908
Female	115 (18.7)	41.4	
Marital status			
Married	344 (55.8)	41.6	0.834
Single and unmarried	131 (21.3)	40.1	
Widowed, divorced, separated	123 (20.0)	49.2	
Unknown	18 (2.9)	41.6	
Race			
White	404 (65.6)	46.4	0.300
Black	89 (14.4)	40.8	
Other	123 (20.0)	33.2	
Tumor size			
≤1 cm	3 (0.5)	33.3	0.022
1‐3 cm	44 (7.1)	59.4	
3‐5 cm	93 (15.1)	44.5	
>5 cm	353 (57.3)	44.6	
Unknown	123 (20.0)	31.5	
Vascular invasion			
Code 630	378 (61.4)	44.1	0.770
Code 635	160 (26.0)	40.7	
Code 660	78 (12.7)	41.0	
TNM/T			
T3	538 (87.3)	43.1	0.587
T4	78 (12.7)	41.0	
Lymph nodes			
N0	441 (71.6)	46.6	<0.001
N1	117 (19.0)	36.2	
Nx	58 (9.4)	28.7	
Distant metastasis			
M0	427 (69.3)	51.5	<0.001
M1	171 (27.8)	22.1	
Mx	18 (2.9)	38.9	
Derived AJCC Stage Group (6th)			
III	427 (69.3)	51.5	<0.001
IV	171 (27.8)	22.1	
Unk stage	18 (2.9)	38.9	
Grade (differentiated)			
Well	58 (9.4)	47.9	0.339
Moderately	76 (12.3)	46.8	
Poorly	51 (8.3)	40.9	
Undifferentiated/anaplastic	2 (0.3)	100.0	
Unknown	429 (69.6)	41.4	
AFP			
Elevated	455 (73.9)	41.0	0.003
Within normal limits	75 (12.2)	61.7	
Unknown	86 (14.0)	36.1	
Fibrosis score			
0‐4 (F0)	32 (5.2)	44.7	0.381
5‐6 (F1)	155 (25.2)	47.9	
Unknown	429 (69.6)	40.9	

AJCC, American Joint Committee on Cancer.

Multivariate predictors of mortality in the propensity‐matched samples are shown in Table 4. Radiation‐treated patients in propensity‐matched sample had a significantly lower risk of mortality (HR: 0.625, 95% CI: 0.522‐0.749, *P* < 0.001) compared with untreated patients, respectively. Distant metastasis (M1) and AJCC Stage IV were independently associated with higher mortality rate (*P* < 0.001 and <0.001, respectively). Patients with elevated AFP levels were associated with higher mortality rate than patients with normal limits.

**Table 4:** Multivariate predictors of overall mortality in the propensity‐matched sample

	Propensity‐matched sample (n = 616)		
	HR	95% CI	*P* value
Radiation (vs untreated)	0.625	0.522‐0.749	<0.001
Tumor size 1‐3 cm (vs ≤1 cm)	1.138	0.344‐3.766	0.832
Tumor size 3‐5 cm (vs ≤1 cm)	1.363	0.426‐4.359	0.602
Tumor size >5 cm (vs ≤1 cm)	1.528	0.484‐4.817	0.47
Code 635 (vs Code 630)	1.006	0.813‐1.246	0.954
Code 660 (vs Code 630)	0.894	0.679‐1.178	0.427
TNM/T4 (vs T3)	0.894	0.679‐1.178	0.427
Lymph nodes N1 (vs N0)	1.153	0.916‐1.451	0.225
Lymph nodes Nx (vs N0)	1.188	0.867‐1.628	0.284
Distant metastasis M1 (vs M0)	1.852	1.508‐2.273	<0.001
AJCC Stage IV (vs III)	1.852	1.508‐2.273	<0.001
Grade moderately (vs Well)	1.275	0.863‐1.883	0.223
Grade poorly (vs Well)	1.407	0.925‐2.139	0.111
Grade undifferentiated/anaplastic (vs Well)	0.597	0.079‐4.523	0.618
AFP within normal limits (vs elevated)	0.671	0.504‐0.895	0.007
Fibrosis score 5‐6 (F1) (vs F0)	1.082	0.695‐1.686	0.727

AJCC, American Joint Committee on Cancer.

### 3.4 Predictors of survival among radiation‐treated patients

Predictors of survival among radiation‐treated patients were evaluated with multivariate analysis, and the results were shown in Table 5. The radiation‐treated single and unmarried patients demonstrated better survival than the married patients (HR = 0.538, 95%CI 0.347‐0.834, *P* = 0.006). The radiation‐treated patients of tumor size >5 cm had significantly worse mortality compared with radiation‐treated patients of tumor size 1‐3 cm (HR 1.836, 95% CI 1.050‐3.211, *P* = 0.033). Overall, the survival among radiation‐treated patients was significantly associated with distant metastasis and AJCC Stage (Both HR: 3.197, 95% CI: 2.287‐4.469, *P* < 0.001). Elevated AFP levels and Fibrosis score F1 were significantly associated with increased risk of mortality (*P* < 0.05).

**Table 5:** Multivariate predictors of survival in radiation‐treated patient (n = 308)

	HR	95% CI	*P*
Age >65 (vs ≤65)	0.999	0.735‐1.358	0.993
Female (vs Male)	1.044	0.731‐1.493	0.811
Single and unmarried (vs Married)	0.538	0.347‐0.834	0.006
Widowed, divorced, separated (vs Married)	1.013	0.702‐1.460	0.947
Black (vs White)	1.019	0.674‐1.540	0.93
Other (vs White)	0.924	0.617‐1.384	0.702
Tumor size 3‐5 cm (vs 1‐3 cm)	1.868	0.970‐3.599	0.062
Tumor size >5 cm (vs 1‐3 cm)	1.836	1.050‐3.211	0.033
Code 635 (vs Code 630)	0.845	0.603‐1.186	0.331
Code 660 (vs Code 630)	1.046	0.667‐1.643	0.844
TNM/T4 (vs T3)	1.046	0.667‐1.643	0.844
Lymph nodes N1 (vs N0)	1.215	0.822‐1.794	0.329
Distant metastasis M1 (vs M0)	3.197	2.287‐4.469	<0.001
AJCC Stage IV (vs III)	3.197	2.287‐4.469	<0.001
Grade moderately (vs Well)	1.183	0.665‐2.105	0.567
Grade poorly (vs Well)	1.418	0.783‐2.569	0.249
Grade undifferentiated/anaplastic (vs Well)	0.606	0.071‐5.189	0.647
AFP within normal limits (vs elevated)	0.577	0.364‐0.915	0.019
Fibrosis score 5‐6 (F1) (vs F0)	2.506	1.107‐5.674	0.028

AJCC, American Joint Committee on Cancer.

### 3.5 Subgroup analysis after PSM

**Figure 3:**
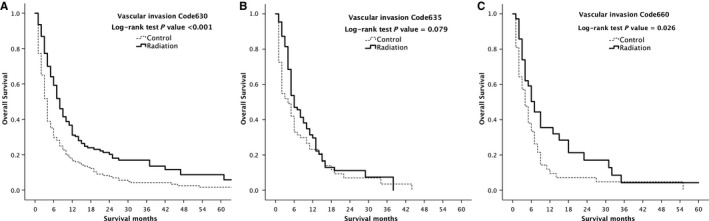
Kaplan‐Meier overall survival curves of radiation‐treated patients vs control after propensity score matching stratified by type of vascular invasion, Code 630 (A), Code 635 (B) and Code 660 (C)

Subgroup analysis was performed on different types of vascular invasions, and the results showed that the survival time of patients with RT in Code 630 and Code 660 subgroup was significantly longer than that in the untreated group (*P* < 0.001 and *P* = 0.026, respectively). The survival rate of patients in the Code 635 group was not significantly affected by RT, but the median survival time was 6 months (95% CI: 4.36‐7.64) in the radiation‐treated group, which was longer than 4 months (95% CI: 2.5‐5.5) in the untreated group (*P* = 0.079, Figure 3).

**Figure 4:**
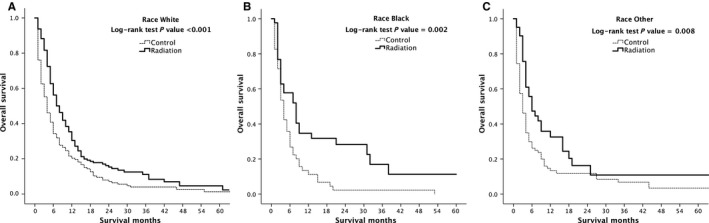
Kaplan‐Meier overall survival curves of radiation‐treated patients vs control after propensity score matching stratified by race white (A), black (B) and other (C)

After PSM, marital status, race, and tumor size still demonstrated group differences, and so further subgroup analysis was performed. Overall survival in radiation‐treated patients after PSM were significantly longer than untreated patients according to subgroup analysis of race white, black, and others (*P* < 0.001, *P* = 0.002 and *P* = 0.008, respectively, Figure 4).

**Figure 5:**
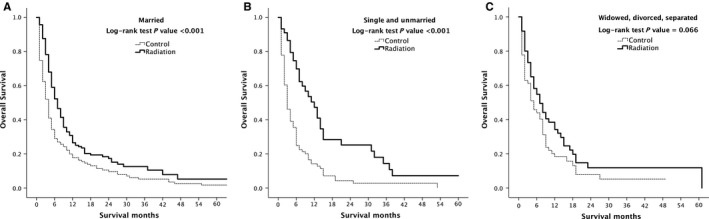
Kaplan‐Meier overall survival curves of radiation‐treated patients vs control after propensity score matching stratified by Marital status married (A), single and unmarried (B), widowed, divorced, separated (C)

In the marital status subgroup, the survival rate of married patients with RT was significantly higher than that in the untreated group (*P* < 0.001). The survival rate of single and unmarried group patients with RT was significantly higher than that in the untreated group (*P* < 0.001). Although there was no significant difference in the survival rate among the widowed, divorced, and the separated group (*P* = 0.066), the median survival time was 7 months (95% CI: 4.267‐9.733) in the RT group, which was higher than 5 months (95% CI: 2.802‐7.198) in the untreated group (Figure 5).

**Figure 6:**
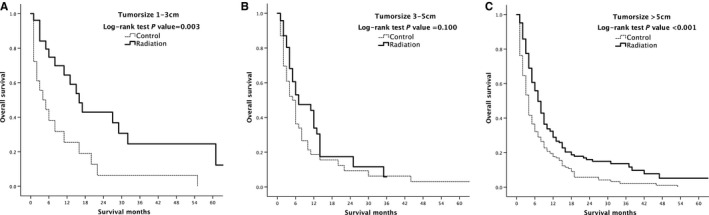
Kaplan‐Meier overall survival curves of radiation‐treated patients vs control after propensity score matching stratified by type of tumor size 1‐3 cm (A), 3‐5 cm (B) and >5 cm (C)

Overall survival of radiation‐treated patients vs control after PSM stratification by tumor size was performed. Patients with tumor size <1 cm did not undergo RT, and so there was no participation in the stratification. According to subgroup analysis, the survival rate of patients with tumor size 1‐3 cm and >5 cm was significantly higher than that of untreated patients (*P* = 0.003 and *P* < 0.001, respectively). There was no significant difference in the survival rate between 3 and 5 cm group of patients, but the median survival was 7 months (95% CI: 1.404‐12.596) in the radiation‐treated patients, which was longer than 5 months (95% CI: 3.233‐6.767) in the untreated patients (Figure 6).

### 3.6 Survival benefits of different RT modality

**Figure 7:**
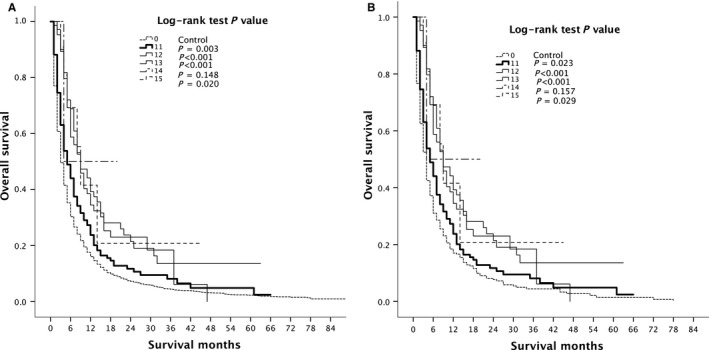
Kaplan‐Meier overall survival curves of different radiotherapy methods treated patients vs control (A) before and (B) after propensity score matching. 0 = Control; 11 = Beam radiation; 12 = Radioactive implants; 13 = Radioisotopes; 14 = Combination of beam with implants or isotopes; 15 = Radiation, NOS method or source not specified

The modality of RT in this article included beam radiation, radioactive implants, radioisotopes, combination of beam with implants or isotopes, and other radiation methods or source not specified. Compared with untreated group, the results showed that the treated group patients with beam radiation, radioactive implants, radioisotopes or other radiation methods or sources not specified had better survival rate before and after PSM and the differences were statistically significant. The radiation combination of beam with implants or isotopes showed no statistical differences (Figure 7). However, there were only three patients who were treated with combination of beam and implants or isotopes. Because of the small sample size, further study is required to confirm these results.

## 4 DISCUSSION

The study showed that the prognosis of HCC patients associated with major vascular invasion was extremely dismal and the median survival time was very short, which was only 3 months in the untreated group. This result was consistent with most of the previous reports.^6^, ^23^, ^24^ Because of low tolerance to whole liver irradiation and concerns about radiation‐induced liver disease (RILD),^25^, ^26^ RT had been used less. Due to the improvement in RT technology and the application of computed tomography over the past decade, radiation can be precisely delivered, and the incidence of RILD can be significantly lowered than before, that is, within the range of acceptable adverse reactions.^27^

The vascular invasion in this article included major branch(es) of portal or hepatic vein(s) and hepatic artery or vena cava. We performed subgroup stratification analysis of vascular invasion. With vascular invasion of codes 630 and 660, the survival rate of patients who underwent RT was significantly improved compared with those of untreated patients. Although there was no significant improvement in the survival of patients in code 635, the median survival time was higher than that of the untreated patients. Vascular invasion of code 635 involves multiple nodules in more than one lobe of liver or on surface of parenchyma. Extent of primary lesion and the location of the lesions also affected the effect of RT.^28^, ^29^ Therefore, vascular invasion codes 630 and 635 are different in this article, because their lesions are different in scope and location, and have an effect on the assessment of RT. For code 635, systemic treatment may be more appropriate, and hence requires more researches to investigate.

At present, most of the literature reports are HCC with portal vein tumor thrombus. Portal vein invasion is also the most common manifestation seen. The hepatic vein/inferior vena cava is less explored, compared with PVTT. It is reported that surgical resection of hepatic vein/inferior vena cava can improve the survival rate of patients.^5^ It has been reported that TACE combined with RT can improve the survival rate of HCC patients with a tumor thrombus in the inferior vena cava and right atrium.^30^ Hepatic artery tumor invasion is quite rare and the number of cases seen till date is few, and so the research reports were relatively less.

We performed subgroup stratification analysis of RT modalities. Almost all the RT modalities significantly improved the survival rate of patients, but this paper did not report adverse reactions. With advances in the imaging technology and further innovative RT technologies, such as three‐dimensional conformal RT (3D‐CRT),^31^ stereotactic body ablative RT (SABR)^32^ and particle beam therapies, it is a pleasure with more precise and less adverse reactions. RT is more widely applied in patients with HCC. In addition, internal RT is gradually taken seriously.

Although there is no clear guidance on the value of RT in advanced HCC, several comparative studies have demonstrated the role of RT in the treatment of HCC patients, including the improvement of survival in HCC patients with vascular invasion.^33^, ^34^ Nakazawa et al compared sorafenib combined with RT for patients with unresectable HCC and with PVTT, and the results showed that the RT group had significantly longer median survival time than sorafenib group (median survival, 10.9 vs 4.8 months; *P* = 0.025).^35^

Some scholars suggested that direct RT for tumor thrombosis can improve the patient's survival rate. Choi et al demonstrated treatment of HCC patients accompanied by PVTT with localized concurrent chemoradiotherapy. RT included both primary tumor and PVTT. The results showed that the overall survival of patients with both primary tumor and PVTT was the longest (median, 16.7 months), survival neither in tumor nor in PVTT was median, 8.4 months, and survival in the tumor alone was median, 16.0 months.^36^ The role of RT in the treatment of HCC should be paid more and more attention. An informal committee of the National Cancer Institute's Radiation Research Program suggested RT as a curative local therapy and should be incorporated into the BCLC staging system, and recommended RT as a palliative treatment for HCC patients with vascular invasion. The committee emphatically recommended combination of RT with regional or systemic therapy for advanced HCC.^28^

Single treatment such as RT or other therapy alone is unsatisfactory for improving the survival. Hence, there is a lot of research going on to explore the common survival benefit of multiple treatments. RT was recommended in combination with regional or systemic therapy, and as a palliative measure. A systematic meta‐analysis showed that the survival rate for TACE plus RT was significantly better than TACE alone**,** 37 A retrospective analysis was performed on HCC patients with main portal vein tumor thrombus (MPVTT) to evaluate the therapeutic effect of the percutaneous transhepatic portal vein stenting and transarterial chemoembolization (PTPVS‐TACE) combined with or without 3‐dimensional conformal RT (3‐DCRT). The study showed that the cumulative survival rate was significantly improved in patients combined with RT. The 360‐day cumulative survival rate with RT was 32.5%, and that without RT was 6.9% (*P* < 0.01).^38^ A randomized trial of an academic tertiary care center from Asan Medical Center compared TACE plus radiotherapy with sorafenib treatment for patients with advanced hepatocellular carcinoma and macroscopic vascular invasion. The result showed that TACE plus radiotherapy was well tolerated and had a higher progression‐free survival rate and less adverse reactions than sorafenib.^39^

Although this was a large sample study and the follow‐up time was sufficiently long enough, SEER data lacked the record of HCC etiology, liver function index, and performance status. BCLC liver cancer scoring system included liver function score and performance status. This article was based on a simple classification of vascular invasion by Collaborative Stage Data Collection System Coding Instructions. However, it was still not detailed enough. For example, HCC invaded the different branches of the portal vein to form a tumor thrombus, which may have an effect on radiotherapy. We hope to refine this section in subsequent forward‐looking articles. Although the database contains specific radiotherapy and surgical treatment methods, the lack of chemotherapy content has a certain impact on the evaluation of radiotherapy, which is the limitation of this manuscript. Therefore, we need more detailed data to evaluate the efficacy and adverse reactions of RT. In addition, this was a retrospective study. Although PSM was used to minimize the selection bias of the radiation‐treated group, other biases that were not considered probably existed. More studies especially randomized controlled trials are required to evaluate the therapeutic effect of RT.

In conclusion, we have shown that RT provides another treatment and improves survival in HCC patients with vascular invasion, especially for tumor(s) confined to one lobe and not on the surface of the liver. RT is an effective local therapy, and combination with other regional or systemic therapies might be a more meaningful measure.

## CONFLICT OF INTEREST

All the authors have no conflicts of interests to declare.
